# Amyloid-beta and phosphorylated tau in post-mortem Alzheimer’s disease retinas

**DOI:** 10.1186/s40478-018-0650-x

**Published:** 2018-12-28

**Authors:** Jurre den Haan, Tjado H. J. Morrema, Frank D. Verbraak, Johannes F. de Boer, Philip Scheltens, Annemieke J. Rozemuller, Arthur A. B. Bergen, Femke H. Bouwman, Jeroen J. Hoozemans

**Affiliations:** 1 Department of Neurology, Alzheimer Center Amsterdam, Amsterdam Neuroscience, Vrije Universiteit Amsterdam, Amsterdam UMC, Mailbox 7057, Amsterdam, 1007 MB The Netherlands; 2 Department of Pathology, Amsterdam Neuroscience, Amsterdam UMC, Vrije Universiteit Amsterdam, Amsterdam, The Netherlands; 3 Ophthalmology Department, VU University Medical Center, Amsterdam, The Netherlands; 4 Department of Physics, Bio Laser Lab Amsterdam, VU University, Amsterdam, The Netherlands; 5 Departments of Clinical genetics and Ophthalmology, Amsterdam UMC, Academic Medical Center, University of Amsterdam, Amsterdam, The Netherlands; 6 The Netherlands Institute for Neuroscience (NIN-KNAW), Amsterdam, The Netherlands

**Keywords:** Retina, Amyloid, Tau, Post-mortem, Alzheimer’s disease, Neurodegeneration

## Abstract

In-vivo labeling of retinal amyloid-beta(Aβ) and tau has potential as non-invasive biomarker for Alzheimer’s disease (AD). However, literature on the presence of Aβ and phosphorylated tau (pTau) in AD retinas is inconclusive. We therefore assessed the presence of Aβ and pTau in post-mortem retinas in 6 AD and 6 control cases who donated brains and eyes to the Netherlands Brain Bank. Neuropathological diagnosis of AD was made according to NIA-AA criteria. Formalin fixed retinas were dissected in quadrants and cross-sections of medial and superior retinas were made. Immuno-histochemical stainings were performed for Aβ, amyloid precursor protein (APP) and pTau. To assess translation to an in-vivo set up using curcumin as labelling fluorophore, co-stainings with curcumin were performed. No typical Aβ-plaques and neurofibrillary tangles, like in the cerebral cortex, were observed in AD retinas. A diffuse immunoreactive signal for pTau was increased in the inner and outer plexiform layers of the retina in AD cases compared to control cases with absence of cerebral amyloid pathology. Immunostaining with anti-Aβ and anti-APP antibodies yielded signal in ganglion cells, amacrine cells, horizontal cells and Müller cells in both control and AD cases. We observed small extracellular deposits positive for anti-Aβ antibodies 12F4 and 6E10 and negative for 4G8 and curcumin. A subset of these deposits could be characterized as corpora amylacea. In conclusion we found that retinal manifestations of AD pathology appear to be different compared to cerebral AD pathology. Using a qualitative cross-sectional approach, we did not find Aβ/APP related differences in the retina between AD and control subjects. In contrast, tau related changes were found to be present in cases with cerebral AD pathology, suggesting retinal tau as a potential biomarker for AD.

## Introduction

Alzheimer’s disease (AD) is the leading cause of dementia [1] and its pathology develops 15–20 years before symptom onset [19]. Key pathological hallmarks of AD are neuronal loss, extra-cellular accumulation of amyloid-plaques containing amyloid-beta (Aβ) and neurofibrillary tangles containing tau [6]. In addition, cerebral amyloid angiopathy (CAA) [33] and neuro-inflammation [13] are recognized as important events involved in AD pathology. The retina, embryologically derived from the diencephalon and sharing many features with brain tissue, is hypothesized to serve as a ‘window to the brain’, as neuropathological changes in retinal neurons might mirror brain pathology [26]. Non-invasive retinal imaging is routinely performed in ophthalmology clinics with high resolution imaging (±5 μm). Fluorescent imaging of specific proteins is on the horizon and might serve as a non-invasive diagnostic tool to visualize molecular processes involved in ophthalmological and neurological disease [5, 7, 22].

Previous post-mortem studies have investigated the presence of pathological changes in human retinas in AD, however the detection of these changes was not consistent across these studies [20]. On the one hand, neuronal thinning, ganglion cell loss and tau deposition are reported [3, 30]. On the other hand, Aβ deposition was found in whole mounted retinas and cross-sections by three studies from one lab, preferentially affecting the superior retina [22–24]. Recently, results from an in-vivo study showed an increase in retinal fluorescence after curcumin supplementation in AD patients, hypothesized to reflect retinal amyloid [22]. However, Aβ presence in the retina still remains controversial as three other groups were unable to replicate this finding in AD retinas [14, 30, 34].

Possible explanations for these discrepancies are differences in methodology, as well as interpretation of findings. First, different anti-Aβ antibodies were used (including 6E10, 12F4, 6F3D and 4G8). Second, different methods of processing and dissection of retinas were applied, as some groups prepared retinal cross-sections while others generated flat mounts. A cross-sectional approach yields morphological information on layer distribution of the pathology, at the cost of sampling bias, due to the limited surface covered. In contrast, using flat mounts, a large surface of the retina is studied, yet lacking layer information. Lastly, with the predilection of pathology in the superior retina reported [22], it might be that other groups assessed parts of the retina with less pathological changes.

To better understand retinal involvement in AD pathology, we explored the presence of AD pathology with different antibodies in post-mortem retinal and brain tissue of patients with a definite post-mortem diagnosis of AD, in order to assess molecular targets for in vivo (fluorescent) retinal imaging in AD.

## Materials and methods

### Subjects

We assessed retinal tissue of 6 AD patients and 6 controls who donated their brain and eyes to the Dutch Brain Bank between 2010 and 2015. Neuropathological diagnosis of AD was made following NIA-AA criteria including Braak and Braak-staging for neurofibrillary tangles and amyloid, and CERAD-staging for neuritic plaques [4, 17]. We selected cases with a clinical diagnosis of AD and neuropathological staging of Braak ≥ 4. Controls were adults without cognitive decline and Braak ≤2. We excluded cases with other neurodegenerative diseases based on history or post-mortem diagnosis. Cohort characteristics are shown in Table 1.

**Table 1:** Cohort characteristics

#	Pathological diagnosis	Sex	Age	Braak	Amyloid
1	AD	F	73	VI	C
2	AD	M	70	VI	C
3	AD	M	56	V	C
4	AD	F	91	V	C
5	AD	F	95	IV	C
6	AD	F	89	IV	C
7	HC	F	89	II	B
8	HC	M	80	II	C
9	HC	F	76	II	O
10	HC	F	79	II	O
11	HC	F	57	0	A
12	HC	M	49	0	O

*AD* Alzheimer’s disease, *HC* Healthy control

### Tissue processing

**Fig. 1:**
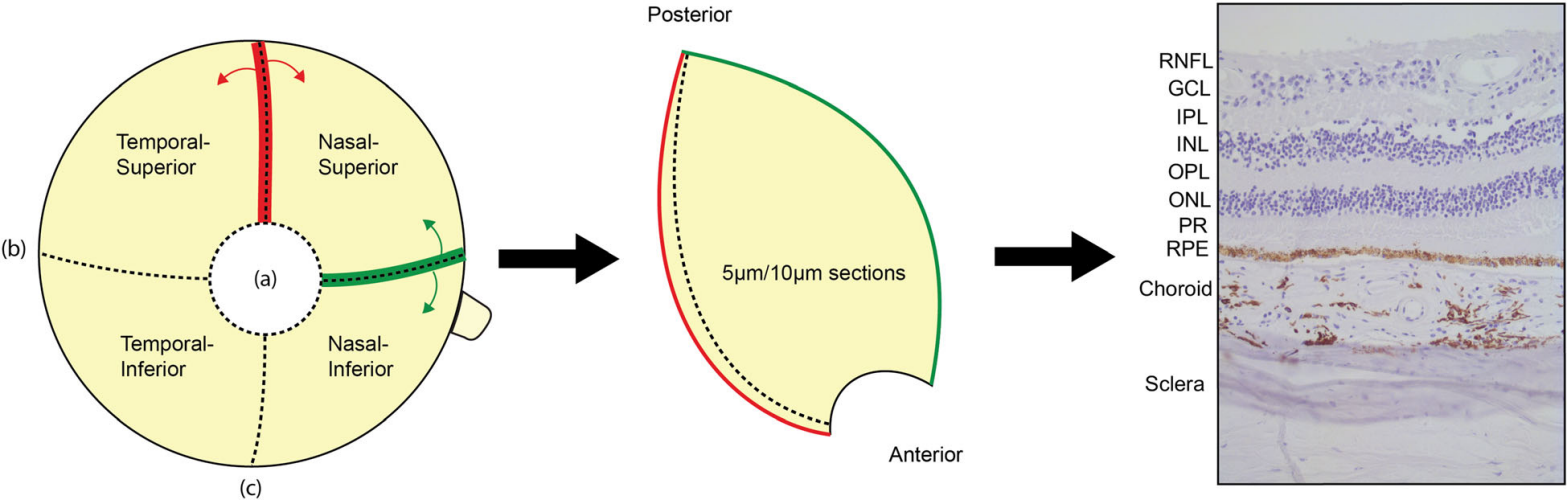
Processing of post-mortem eyes. Anterior parts of eyes were removed (a). Formalin fixed eyes were dissected through the horizontal (b) and vertical meridian (c). Superior (red, arrows) and nasal (green, arrows) parts were cut in 10 μm sections from anterior to posterior. As a result, sections contained all retinal layers from ora serrata to the posterior pole: retinal nerve fiber layer (RNFL), ganglion cell layer (GCL), inner plexiform layer (IPL), inner nuclear layer (INL), outer plexiform layer (OPL), outer nuclear layer (ONL), photoreceptors (PR), retinal pigment epithelium (RPE), choroid and sclera

Within 12 h post-mortem, eyes were removed. The anterior parts of the eye, including the cornea and lens, were dissected and the eyecup was filled with tissue-tek (cat# 4583, Sakura). Eyes were snap frozen using iso-pentane at − 100 °C and stored at − 80 °C. Eyes were defrosted in 4% PFA at room temperature for 48 h prior to dissection. The eye was dissected in four quadrants through the vertical and horizontal meridian resulting in naso-superior, naso-inferior, temporal-superior and temporal-inferior quadrants containing retinal tissue from macula to ora serrata (Fig. 1). Quadrants were dehydrated prior to embedding in paraffin according to the following protocol: 3 h formalin 4% at 35 °C, 1 h ethanol 70% 35 °C, 1 h ethanol 80% 35 °C, 1 h ethanol 96% 35 °C, 3 times 1 h alcohol 100% 35 °C, 3 times 1 h xylene 35 °C, 4 times 1 h paraffin 62 °C. Paraffin embedded tissue was sectioned using a microtome at 5-μm and 10-μm thickness and mounted on TOMO slides (cat# TOM-1190, Matsunami). Mounted slides were dried overnight at 37 °C prior to staining. Per patient, at least 25 sections per region were stained with APP/Aβ antibodies to overcome sampling bias.

### Immunohistochemistry (IHC)

Immunohistochemistry was initially performed on 5 and 10 μm thick eye sections. As both thicknesses yielded comparable results, 10 μm sections were therefore used for the full cohort. Sections were deparaffinized and endogenous peroxidase activity was suppressed with 0.3% H_2_O_2_ in phosphate buffered saline (PBS) for 30 min. Antigen retrieval was performed with 10 mM/L pH 6.0 sodium citrate buffer heated by autoclave. Sections were incubated overnight at room temperature with primary antibody, diluted in antibody diluent (cat# kpxxabb500, immunologic). For primary antibodies details and dilutions, see Table 2. Omission of the primary antibodies was taken along as negative controls. Positive controls consisted of 5 μm thick paraffin sections of hippocampal regions of AD patients. Sections were incubated for 30 min with envision (cat# 5007, DAKO). 3,3′-Diaminobenzine (DAB) was used for color development. Nuclear counterstaining consisted of Mayers hematoxylin. Sections were dehydrated and cover slipped using quick-D (cat# 7281, Klinipath).

**Table 2:** Primary antibodies used in this study

Group	Name	Epitope	Source	Concentration
APP/Aβ	12F4	Amyloid-beta_1–42_	Biolegend San Diego, CA, USA	1:100
	6E10	Amyloid-beta_1–16_	Covance, BioLegend, San Diego, CA, USA	1:800
	4G8	Amyloid-beta_17–24_	Biolegend, San Diego, CA	1:8000
	APP	APP Y188, c-terminal (aa 750)	ABCAM, Cambridge, UK	1:6000
Tau	HT7	Tau between residue 159 and 163	Invitrogen, Thermo Fisher Scientific, MA, USA	1:8000
	AT-270	Tau phosphorylated at Thr 181	Thermo Scientific, MA, USA	1:100
	AT8	Tau phosphorylated at Ser 202, Thr205	Innogenetics, Ghent, Belgium	1:800
	AT-100	Tau phosphorylated Ser 212, Thr 214	Thermo Scientific, MA, USA	1:1000
	Anti-pS422	Tau Fibrils	ABCAM, Cambridge, UK	1:400
	MC-1	Paired helical filaments	Peter Davies, Pathology and Neuroscience, Donald and Barbara Zucker School of Medicine, Northwell, NY, USA [21]	1:1000
Other	Curcumin	Fibrillar Aβ and CAA	Sigma Aldrich, St. Louis, MO, USA	0,1 mg/ml in NaOH
	Thioflavin	Amyloid	Sigma Aldrich, St. Louis, MO, USA	1% in distilled water
	Kluver-PAS	Polysaccharides	Kluver: Luxol fast blue, BDH laboratory supplies, Avantor, PA, USA, PAS: Schiff’s reagens, VWR, Avantor, PA, USA	0.1% in alcohol 96% n.a.

Alternatively, when liquid permanent red (LPR) was used as chromogen, sections were incubated for 30 min with goat anti-mouse alkaline phosphatase (AP) (1:250, cat# d0456, DAKO) at room temperature, after incubation with the primary antibody. After washing 3 times with TRIS buffered saline (TBS) sections were incubated with LPR (cat#0640, DAKO), substituted with 1 mM levamisole (to block endogenous alkaline phosphatase from blood vessels), until signal could be observed microscopically. Nuclear counterstaining was performed using Mayer’s hematoxylin. Section were cover-slipped using aquatex (cat#1.08562.0050, Merck).

### Curcumin

Curcumin (cat# 9386, Sigma Aldrich) was dissolved in 0.5 M NaOH to a stock concentration of 20 mg/ml and diluted to a final concentration of 0.1 mg/ml curcumin using PBS. Deparaffinized and rehydrated sections were incubated with 0.1 mg/ml curcumin for 10 min at room temperature, washed with PBS for 3 times 10 min and covered using TBS/glycerol mounting medium.

### Thioflavin-S

Thioflavin-S staining was performed by incubating deparaffinized and rehydrated sections with 1% thioflavin-S (cat# T1897, Sigma Aldrich) in distilled water for 1 min, followed by removal of excess thioflavin-S using 70% alcohol. Stained sections were covered using TBS/glycerol mounting medium.

### Kluver-PAS

Deparaffinized and rehydrated sections were incubated with 0.1% luxol fast blue (cat# 3404438, BDH laboratory supplies) overnight at 37 °C. Sections were subsequently rinsed in ethanol 96% and distilled water. Differentiation was performed by rinsing the slides in 0.1% lithium-carbonate, followed by 70% ethanol and distilled water. Subsequently, the sections were incubated with 0.8% periodic acid for 10 min, followed by rinsing in tap water and distilled water. Sections were incubated with Schiff’s reagent (cat# 30969.261, VWR) for 30 min, followed by washing in tap water. Kluver-PAS stained sections were counterstained using Mayer’s hematoxylin, quickly dehydrated using alcohol and xylene and covered using quick-D.

### PAS-IHC double staining

Deparaffinized and rehydrated sections were incubated and DAB stained as described above. DAB stained sections were washed in distilled water and incubated with 0.8% periodic acid for 10 min. Subsequently, sections were incubated with Schiff’s reagent for 30 min, washed with tap water and counterstained using Mayer’s hematoxylin. Stained sections were quickly dehydrated using ethanol and xylene and covered using quick-D.

### Fluorescent co-stainings with curcumin

Sections were deparaffinized and treated with citrate buffer as described above. Subsequently, curcumin staining was performed as described above. After curcumin staining, sections were washed 3 times for 10 min using PBS. Primary antibody was incubated overnight at room temperature. Sections were washed 3 times with PBS and incubated with fluorescently-labelled secondary antibody (1:250, alexa 594, cat# A11037, Invitrogen) for 3 h. After labelling the sections were washed in PBS and covered using DAPI containing mounting medium (cat# 0100–20, Southern Biotech).

### Imaging

IHC sections were imaged by an Olympus BX41 photomicroscope using Leica Application Suite (LAS) AF lite software (Wetzlar, Germany). Fluorescently stained sections were imaged by a Leica DMi8 fluorescence microscope using LAS AF lite and ImageJ software (National Institute of Health, USA). Spectra of the Kluver-DAB double stainings were analysed using a nuance spectral imager (Perkin Elmer). Colors were assigned to binary spectra images using ImageJ.

### Quantification tau immunohistochemistry

For quantitative assessment sections were photographed at four regions of interest (ROI’s) from ora serrata to posterior pole of medial and superior cross-sections for each patient using an Olympus BX41 photomicroscope. Dimensions of ROI’s were 316 μm × 115 μm. The DAB positive surface area (%) within the neuroretina was quantified using the color threshold plugin in ImageJ. Between group differences were assessed using a Mann-Whitney U test, using SPSS (version 22.0).

## Results

### Immunohistochemical detection of amyloid-beta and APP in the retina

**Fig. 2:**
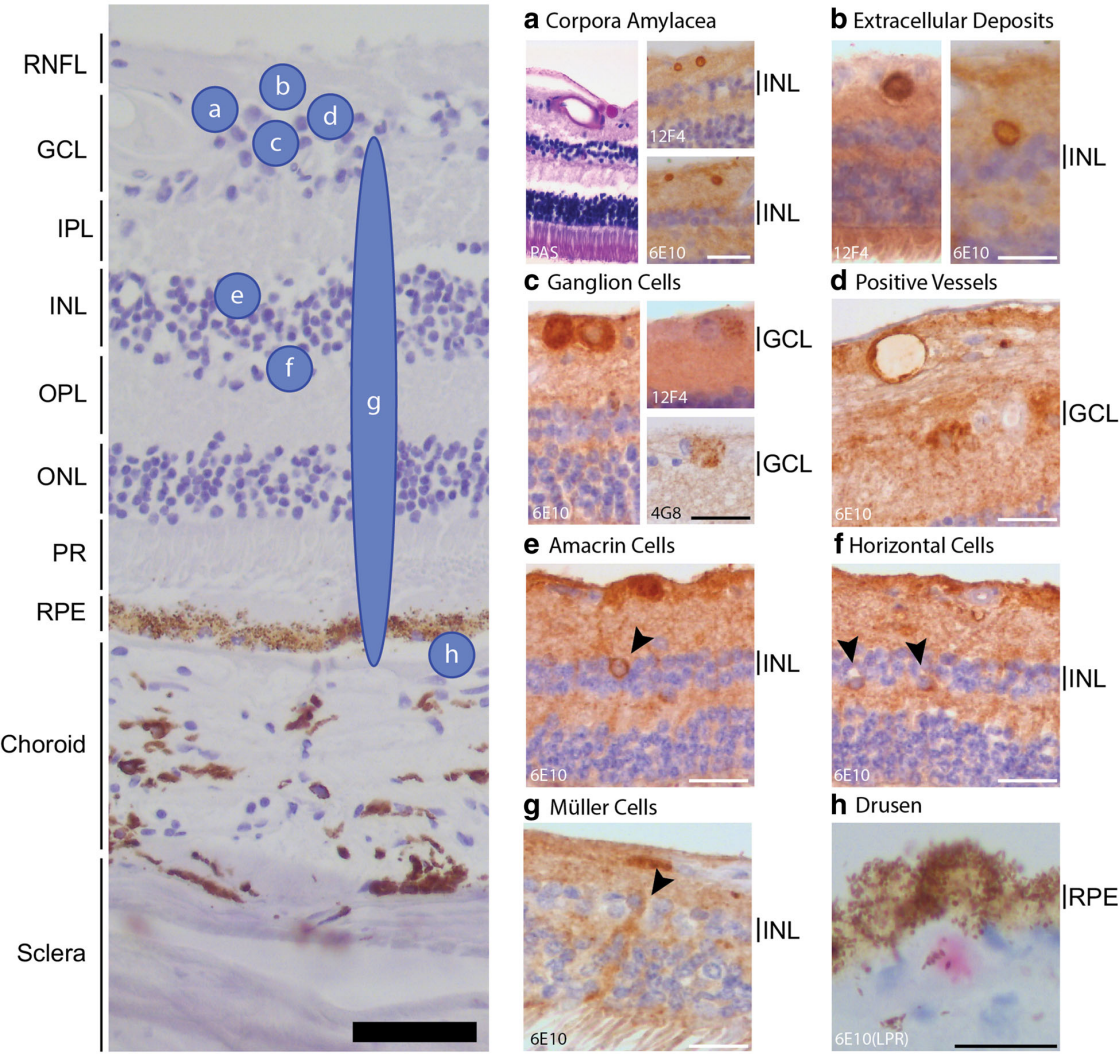
Overview of different structures positive for different anti-Aβ antibodies. Overview of structures that were stained with anti-Aβ antibodies 6E10,12F4 and 4G8. Distribution of these structures over retinal layers is shown on the left (**a**-**h**). Abbreviations: RNFL = retinal nerve fiber layer, GCL = ganglion cell layer, IPL = inner plexiform layer, INL = inner nuclear layer, OPL = outer plexiform layer, ONL = outer nuclear layer, PR = photo receptors, RPE = retinal pigment epithelium, LPR = liquid permanent red. Scale bars 50 μm

**Fig. 3:**
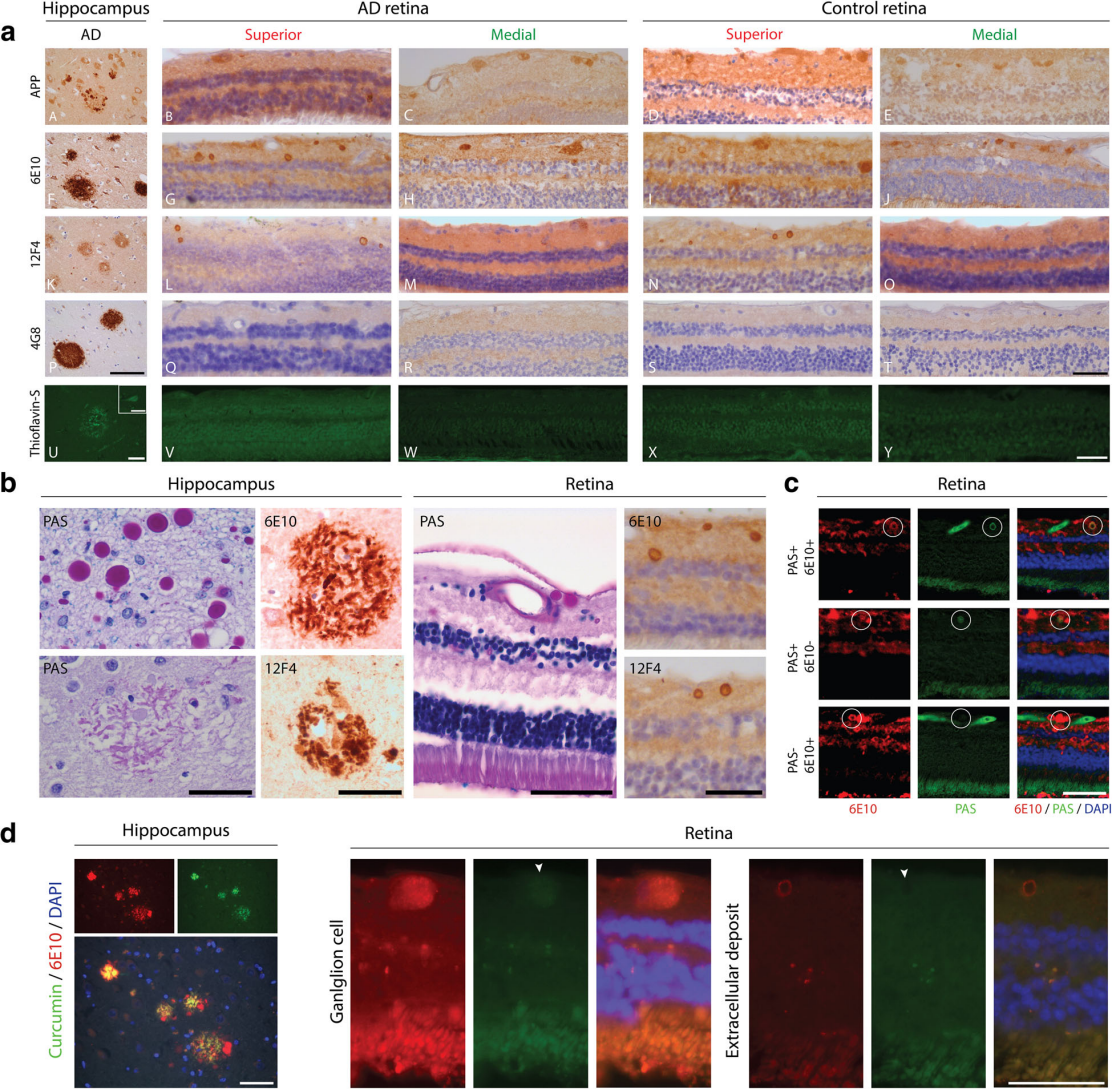
APP/Aβ in the medial and superior retina. **a** Representative stainings for APP/Aβ antibodies and amyloid (Thioflavin-S) in AD hippocampus and AD and control retinas showing different staining patterns for different antibodies (superior and medial). **b** Kluver-PAS staining of corpora amylacea in hippocampus and retina compared to 6E10 and 12F4 stainings. **c** IHC co-stainings with PAS and 6E10. **d** Curcumin and 6E10 co-staining in AD hippocampus and in retinas showing a positive ganglion cell and extracellular deposit. All size bars 50 μm

Medial and superior retinal cross-sections were immunostained for Aβ-plaque pathology using anti-Aβ antibodies 6E10 and 12F4. Retinas stained with 6E10 showed intracellular positivity in specific retinal cell-types including ganglion cells (Fig. 2c), cells in the upper and lower portion of the inner nuclear layer (INL), most likely reflecting amacrine- and horizontal cells respectively (Fig. 2e,f), and Müller cells (Fig. 2g). In addition, staining in vessel walls (Fig. 2d) and in drusen was observed (Fig. 2h). Small extracellular deposits (5–20 μm) were incidentally seen in the ganglion cell layer (GCL) and inner plexiform layer (IPL), with a predilection for the superior retina (Fig. 2b and Fig. 3a). These deposits were small (5-20 μm), often rounded and were morphologically different from cored plaques, diffuse plaques or fibrillary plaques observed in AD brain tissue. Retinas immunostained with 12F4 showed intracellular staining of ganglion cells as well (with a granular aspect), staining in vessel walls and immunostaining of small extracellular deposits in the GCL and IPL, similar as observed with 6E10 staining (Fig. 3a). Above described immunopositive features using antibodies 6E10 and 12F4 were more apparent in the superior part of the retina then in the medial part. Global assessment of the above described features in cross-sections of 6 control and 6 AD cases did not separate AD from control cases (Fig. 3a).

In addition to Aβ, the antibody 6E10 also detects full length APP [16, 35]. Using an antibody directed against APP (C-terminal amino acid (aa) 750), similar staining as compared with 6E10 was seen in ganglion cells, amacrine cells, horizontal cells and Müller cells (Fig. 3a), but not in extracellular and vascular deposits. Subsequently, as extracellular and vascular positivity was not observed after APP immunostaining, we expanded our stainings with anti-Aβ antibodies with less affinity for APP. Anti-Aβ antibody 4G8 showed no extracellular positivity or positivity in blood vessels in AD or control retinas (Fig. 3a). Incidentally, granular staining of ganglion cells was observed with 4G8 (Fig. 2c). Lastly, additional staining with Thioflavin-S, did not show positive staining, while plaques and tangles were observed in AD hippocampal tissue (Fig. 3a).

In summary, intracellular staining was observed with 12F4 and 6E10 and APP antibodies. In addition, 6E10 and 12F4 antibodies showed small extracellular deposits that were negative for 4G8 and APP antibodies.

### Corpora amylacea are detected in AD and control retina

We investigated whether the small 6E10/12F4 positive, 4G8 negative deposits, could be related to corpora amylacea, deposits associated with aging as well as neurodegeneration [29]. We therefore assessed these deposits using a combination of 6E10 antibody and Kluver-PAS staining, a histochemical staining routinely used for the detection of corpora amylacea. With the Kluver-PAS we observed staining of corpora amylacea in AD brain tissue, while plaques were not or only faintly labeled (Fig. 3b). Retinas of both AD and controls showed extracellular, rounded depositions with the Kluver-PAS staining, that were comparable in size and localization to the structures observed with the 6E10 and 12F4 antibodies in adjacent retinal cross-sections (Fig. 3b).

Next we performed double-labelling combining the Kluver-PAS staining with immunodetection with the 6E10 antibody. A subset of 6E10 positive deposits co-localized with Kluver-PAS positive deposits, while others were Kluver-PAS negative (Fig. 3c). In addition, we also observed PAS positive, 6E10 negative deposits (Fig. 3c).

To assess fibrillarity of extracellular deposits and enable translation of our findings to in-vivo imaging with curcumin, we performed Thioflavin-S and curcumin stainings of adjacent sections [8]. No extracellular deposits were detected with curcumin or Thioflavin-S (Fig. 3a and d). This finding was confirmed by fluorescent co-stainings with 6E10 and curcumin (Fig. 3d). Fluorescent co-stainings with 6E10 and curcumin did show co-localization of (faint) curcumin staining with 6E10 intracellularly (Fig. 3d).

In summary, we observed corpora amylacea in AD and control retina that explained a proportion of small 6E10 immunopositive extracellular deposits. These extracellular deposits were negative for Thioflavin or curcumin.

### Tau and phosphorylated tau in AD and control retina

**Fig. 4:**
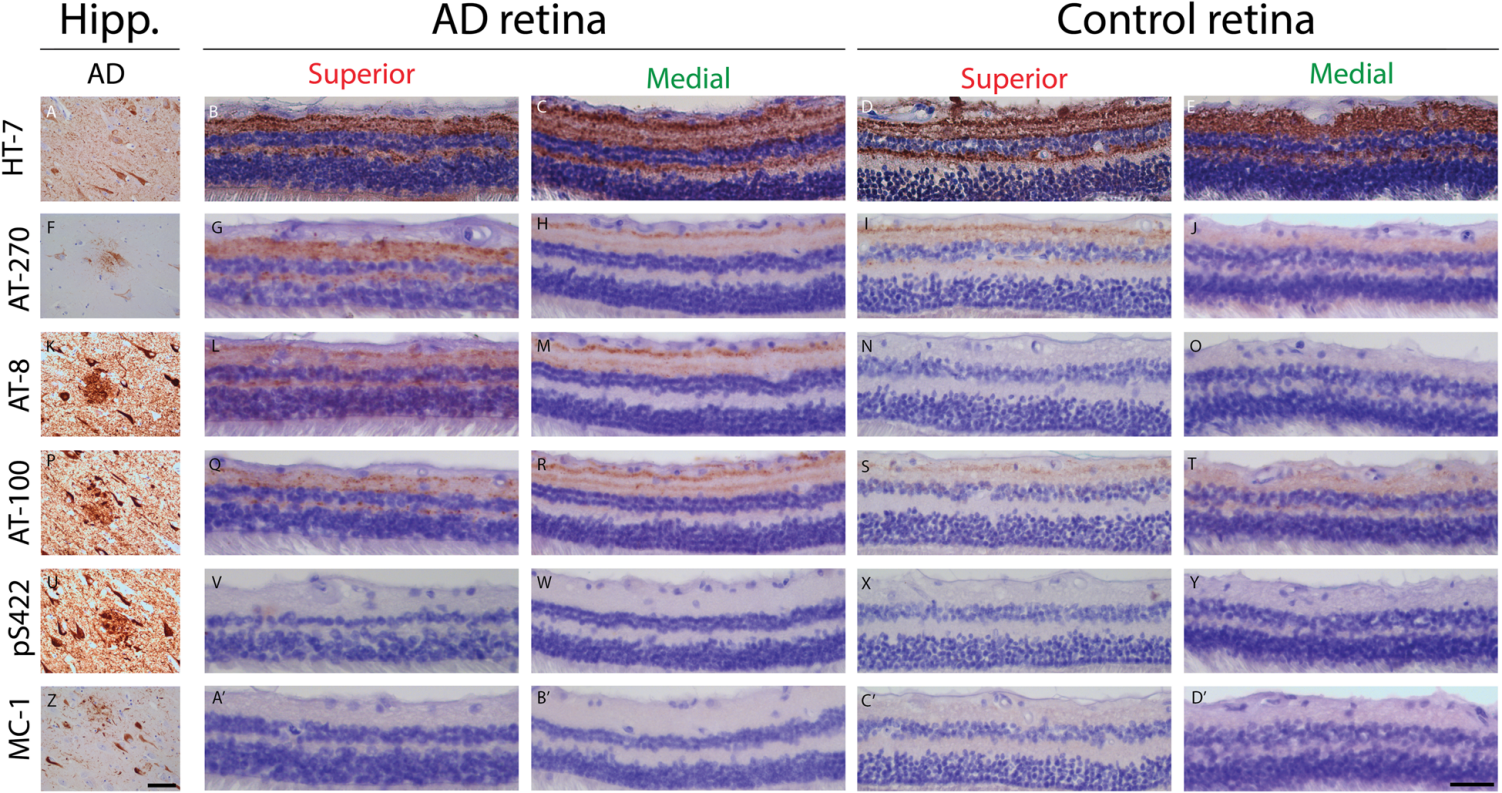
(Phosphorylated) tau in AD retinas. Representative stainings of different tau antibodies in AD hippocampus and AD and control retinas (superior and medial). Antibodies are associated with total tau (HT-7), phosphorylated tau (AT-270, AT-8, AT-100), tau fibrils (anti-pS422) and paired helical filaments (MC-1). Size bars: 50 μm. Hipp. = hippocampus

**Fig. 5:**
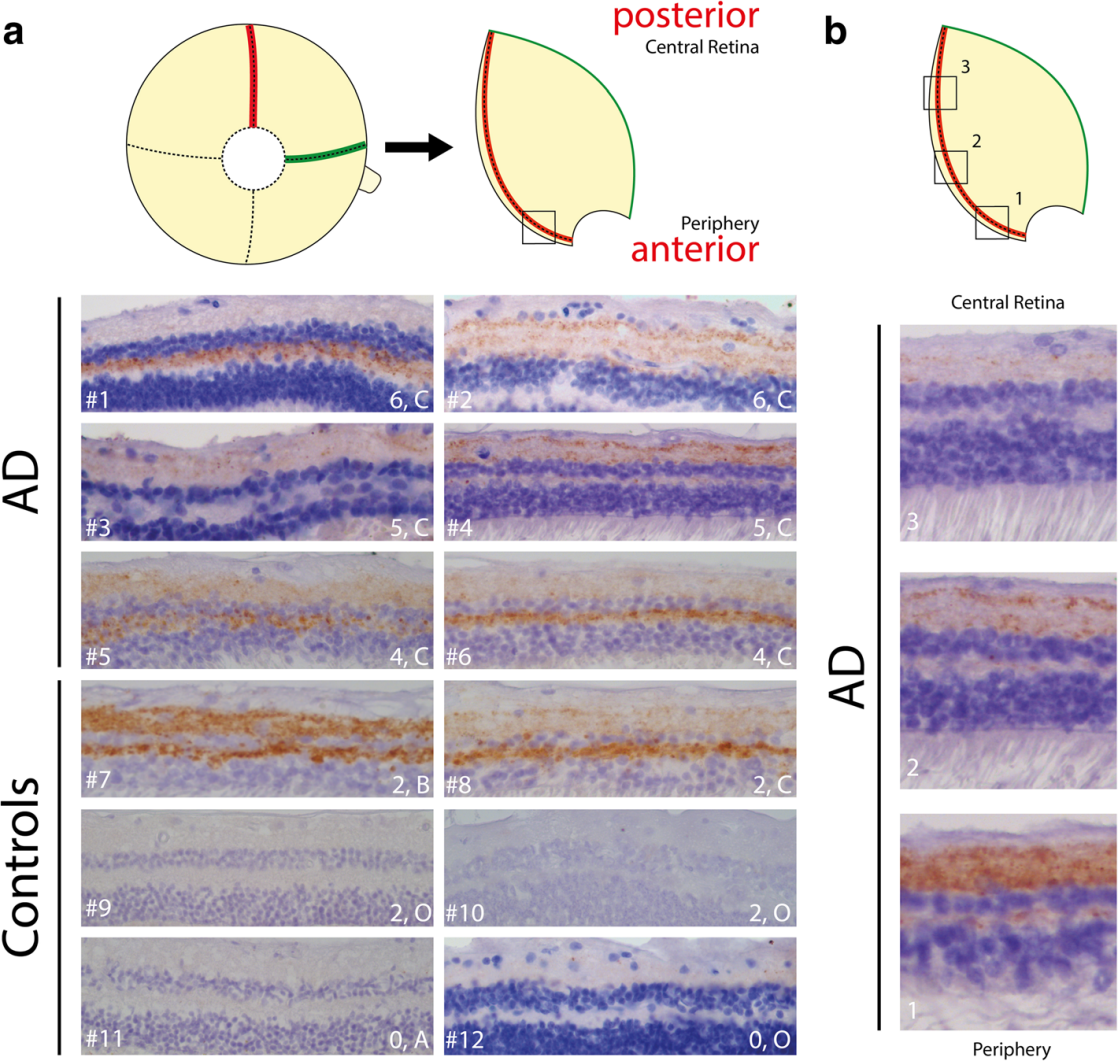
Phosphorylated tau in AD and control retinas. **a** Representative pictures of phosphorylated tau (AT8) stainings of the anterior part of superior retinas for each subject. Case numbers are indicated on the left and Braak Tau and Braak Amyloid stage are indicated on the right. **b** Positive gradient of phosphorylated tau staining towards the periphery in a representative AD case (#4)

Next to amyloid pathology, AD is characterized by tau pathology, which encompasses accumulation of phosphorylated tau (pTau) in neuropil threads, neurofibrillary tangles (NFTs) and neuritic plaques, primarily in cortical areas of the brain [31]. We therefore assessed presence of tau and its phosphorylated isoforms in AD and control retinas. As reported previously [25], total tau assessed with antibody HT7 shows tau distribution in three distinct layers in the inner plexiform layer (IPL) and diffusely in the outer plexiform layer (OPL) (Fig. 4). Diffuse axonal positivity in IPL and OPL was observed with anti-pTau antibodies AT8, AT100 and AT270 (Fig. 4). Overall, more immunoreactivity for AT8, AT100 and AT270 was present in AD cases compared to controls (Fig. 5). However, no structures related to NFTs, neuropil threads or neuritic plaques, as observed in the cerebral cortex, were observed with these antibodies in AD or control retinas. pTau (AT8) staining was more apparent in superior than in medial parts of the retina and showed a positive gradient towards the periphery (Fig. 5). Two controls with an amyloid Braak stage B and C showed similar pTau (AT8) patterns as observed in the retina of AD cases. Table 3 shows quantification of AT8 immunoreactivity in medial and superior retinas of AD versus control cases. Mean surface of AT8 immunopositivity in medial and superior parts of the retina did not statistically differ between AD cases and controls (Mann-Whitney U test *p* = 0.310 and *p* = 0.537 respectively), as two controls with moderate to severe amyloid pathology were considerable outliers. Stratified for amyloid stage, cases with a moderate to severe amyloid stage (B,C) showed higher AT8 immunopositivity compared to cases with low amyloid stages (O, A) in medial (Mean 5.28% (±9.83) vs. 0.01%(±0.01), Mann-Whitney U test, *p* = 0.004) and superior parts of the retina (Mean 12.14%(±15.60) vs. 0.06%(±0.08), Mann-Whitney U test, *p* = 0.012). No immunostaining in control and AD retina was observed with anti-tau pS422 or antibody MC-1, which specifically detects paired helical filaments (Fig. 4).

**Table 3:** Quantification of phosporylated Tau (AT8) Positivity

#	Pathological diagnosis	Braak Tau	Braak Amyloid	AT8 positivity (% surface area)			
				Superior		Medial	
				Mean	sd	Mean	sd
1	AD	VI	C	3,41	2,82	1,88	0,44
2	AD	VI	C	0,79	0,70	1,07	0,55
3	AD	V	C	1,35	0,38	0,04	0,05
4	AD	V	C	15,36	5,13	4,12	2,52
5	AD	IV	C	6,87	1,02	0,37	0,01
6	AD	IV	C	8,72	1,65	1,97	1,25
			Mean	6.08	5.50	1.57	1.47
7	HC	II	B	48,65	8,73	29,35	2,62
8	HC	II	C	11,92	1,84	3,43	1,87
9	HC	II	O	0,15	0,18	0,00	0,01
10	HC	II	O	–	–	0,01	0,01
11	HC	0	A	0,02	0,02	0,03	0,02
12	HC	0	O	0,01	0,01	0,00	0,00
			Mean	12.15	21.04	5.50	11.78

Means and standard deviations (sd) of AT8 positivity (% surface area) of four superior and medial pictures per patient. *AD* Alzheimer’s disease, *HC* Healthy control

In summary, diffuse phosphorylated tau for three phosphorylation sites was observed in AD, with a predilection for the peripheral retina, while NFTs, neuritic plaques, fibrillar tau or paired helical filaments were not detected.

## Discussion

In this post-mortem study of well-characterized AD and control cases, we qualitatively assessed antibody panels for APP, Aβ and tau on AD and control retinal cross-sections. We found that diffuse phosphorylated tau in the retina separated AD cases from controls while immunoreactivity for APP and Aβ in the retina did not differ between groups.

In order to resolve discrepancies between studies reporting retinal Aβ we, for the first time, assessed the presence of APP and Aβ in the retina using a wide panel of antibodies. Our results implicate that APP/Aβ pathology in the retina does not clearly separate AD cases from controls. Using an APP antibody we showed that a large proportion of immunostaining with 6E10 and 12F4 showed overlap with, and could be explained by, intracellular staining of APP in retinal cell types that are known to express APP [28]. In addition, it could also represent intraneuronal β-secretase cleaved APP β-C-terminal fragments (CTFs) including C83, C99 and AICD [11], as our APP antibody binds to the C-terminal of APP (aa750). We hypothesize that intracellular APP/Aβ might reflect metabolic activity in different retinal cell populations expressing APP, as reported for ganglion cells and INL cells [28], and (efficient) processing of APP/Aβ to the outer retina by Müller cells, cells that are responsible for retinal homeostasis [32]. High levels of APP in the absence of clear extracellular fibrillar Aβ deposits suggests that the amyloidogenic and non-amyloidogenic pathway are differently controlled in the retina, compared to the brain. In addition, the build-up of fibrillar Aβ might be confined to the intracellular compartment in the retina. Assessment of mechanisms controlling the amyloidogenic and non-amyloidogenic pathways in the retina is therefore needed to assess the precise role of Aβ processing in the retina in AD and aging. Understanding these mechanisms the role of retinal amyloid as non-invasive biomarker, but could also yield information on selective vulnerability or resilience of specific neuronal populations [9].

We replicated earlier findings of small extracellular deposits positive for 6E10 and 12F4 antibodies in the RNFL and GCL with a predilection to the superior retina [22]. Morphologically these deposits were different from Aβ-plaques observed in the cortex and hippocampus, smaller and also observed in control cases. Using a wide panel of anti-Aβ and APP antibodies we further characterized these deposits, considering cross-reactivity towards Aβ and APP by these antibodies [16]. Interestingly, observed deposits were negative for an anti-Aβ antibody with relatively less affinity for APP (4G8). In addition, an anti-APP antibody directed at the C-terminal (aa 750) did not show immunoreactivity towards these extracellular deposits. As 6E10 is directed to Aβ_3–10_ and has affinity for APP, a possible explanation is that 6E10 recognizes an APP isoform that is not detected by the used APP antibody, for instance the soluble APPα isoform that lacks the C-terminal part of APP. Extracellular immunopositivity with the 12F4 antibody could be explained by detection of IgMs by 12F4 as described in previous literature [2]. Using IHC co-stainings with Kluver-PAS, we showed that a proportion of these deposits can be characterized as corpora amylacea, deposits previously associated with aging and neurodegeneration in the brain [29]. Finally, the absence of co-staining of these deposits with curcumin and Thioflavin suggests absence of fibrillar structures, like fibrillar Aβ [8], and questions whether these deposits can be visualized in-vivo using curcumin [22].

Secondly, we assessed the presence of tau, its phosphorylated isoforms, fibrillar tau (pS422) and paired helical filaments (MC-1). Using antibodies AT8, AT100 and AT270 directed at different phospho-epitopes of tau revealed a diffuse signal in the inner plexiform layer (IPL) and outer plexiform layer (OPL) of AD cases, while NFT’s, neuropil threads and neuritic plaques were not observed. In addition, we observed no fibrillar tau or paired helical filaments. A previous report by Schön and coworkers also showed a diffuse signal of pTau with AT8, while phosphorylation sites detected by AT100, AT180 and AT270 yielded negative results [30]. In addition, similar to our study no fibrillary tau was observed, while in contrast to our findings, intracellular inclusions (NFTs) were observed in 5 out of 6 AD cases using the AT8 antibody. Discrepancies with this study may be explained by differences in antibody dilutions, retinal regions studied and differences in expression of retinal pathology between AD patients.

The diffuse signal of pTau observed in the current study was most apparent in the peripheral retina, as reported previously in tau transgenic mice [15]. This, might be of interest for in-vivo studies that currently mainly focus on imaging of the central retina. Recent developments in imaging technology make it possible to perform in-vivo peripheral imaging of the retina. This may be a promising step forward in visualizing AD pathology in the peripheral retina [27].

Interestingly, two cognitively normal controls with moderate amyloid pathology showed intense tau staining patterns similar to AD cases. This might suggest early involvement of tau-pathology in the retina in AD. Mechanistically, this could be the result of local pathology or transneuronal propagation of phosphorylated tau originating in the visual cortices [10]. Supporting the latter, both control cases had moderate to severe amyloid pathology in their visual cortices that could have already contributed to local toxicity to dendrites and subsequent tau phosphorylation [18]. On the other hand, caution is warranted for the (over)interpretation of these findings, as tau phosphorylation might also be the result of confounding (retinal) pathology, including glaucoma [12] or an aging effect. Supporting the latter, we observed that a young onset AD case (case #4, 56 years) showed little pTau immunopositivity, while two older controls (case #7 89 years, case #8 80 years) showed apparent diffuse pTau immunopositivity. Future studies in larger cohorts, including patients with a range of Braak and amyloid stages, and considering age as confounder, are needed to assess pTau as a possible AD biomarker in the retina. Studies on different tauopathies are needed to assess the specificity of these findings and the possible use of retinal tau as biomarker in other tauopathies and glaucoma. Finally, development of fluorescent labels for phosphorylated tau for human use is needed in order to translate these findings to an in-vivo set-up using targeted fluorescence. A recent proof of concept study showed the possibility of molecular imaging using targeted fluorescence, by in-vivo labeling of single-cell apoptosis in glaucoma [5].

Important strengths of this study are the use of well-characterized cases and controls, and the use of antibody panels on sequential cross-sections, permitting a qualitative assessment of retinal AD pathology and its layer distribution. Despite the fact that we aimed to address sampling bias by staining a large number of 10 μm thick sections per patient, we should note that we might have missed infrequent pathology, including the temporal and inferior retinal regions. Using flat-mounts in future studies to complement a cross-sectional approach could help overcome sampling bias. Using such an approach, inherently lacking qualitative layer information, caution should be taken that 6E10 and 12F4 positivity includes immunoreactivity towards intracellular APP, corpora amylacea and drusen. Future studies are needed to assess if (peripheral) drusen could represent an increased Aβ reservoir in aging and AD. In addition, for this study a relatively small sample size was assessed using a qualitative approach to assess neuropathological hallmarks of AD in the retina. Disease effects are typically observed in comparable cohorts. However, to further understand observed effects and study whether more subtle changes are present in the retina larger cohorts should be assessed using a quantitative approach.

## Conclusion

Our findings confirm the presence of phosphorylated tau in the retina, nuance earlier findings of retinal Aβ and explain negative findings from previous studies, using antibodies with less APP affinity. Independent studies using comparable protocols for tissue processing and staining are needed to resolve discrepancies between different labs [14, 22, 30, 34]. These studies could help determine which of the observed retinal changes are age-related and which are disease related and could serve as a target for in-vivo imaging. In conclusion, neuropathological hallmarks of AD appear differently in the retina compared to the cerebral cortex. Diffuse phosphorylated tau in the peripheral retina might be a promising target for in-vivo imaging.

## Abbreviations

°C: Celsius
AD: Alzheimer’s disease
APP: Amyloid precursor protein
Aβ: Amyloid-beta
CAA: Cerebral amyloid angiopathy
CERAD: Consortium to establish registry for Alzheimer’s disease
DAB: 3,3′–diaminobenzidine
DAPI: 4′6-diamidino-2-phenylindole
GCL: Ganglion cell layer
IHC: Immunohistochemistry
INL: Inner nuclear layer
IPL: Inner plexiform layer
LPR: Liquid permanent red
NBB: The Netherlands brain bank
NFTs: Neurofibrillary tangles
NIA-AA: National Institute of Aging and Alzheimer’s Association
OPL: Outer plexiform layer
PAS: Periodic acid Schiff
PBS: Phosphate-buffered saline
PFA: Paraformaldehyde
PR: Photo receptors
pTau: Phosphorylated tau
RNFL: Retinal nerve fiber layer
RPE: Retinal pigment epithelium
TBS: Tris-buffered saline
